# Genetic and immunologic findings in children with recurrent aphthous stomatitis with systemic inflammation

**DOI:** 10.1186/s12969-021-00552-y

**Published:** 2021-05-10

**Authors:** Martina Girardelli, Erica Valencic, Valentina Moressa, Roberta Margagliotta, Alessandra Tesser, Serena Pastore, Ottavia Spadola, Emmanouil Athanasakis, Giovanni Maria Severini, Andrea Taddio, Alberto Tommasini

**Affiliations:** 1 Institute for Maternal and Child Health, IRCCS Burlo Garofolo, Trieste, Italy; 2 University of Trieste, Trieste, Italy

**Keywords:** Recurrent aphthous stomatitis, Behçet’s disease, Systemic Lupus Erythematosus, Interferon signature, A20 haploinsufficiency, *STAT1* mutation

## Abstract

**Background:**

Recurrent aphthous stomatitis with systemic signs of inflammation can be encountered in inflammatory bowel disease, Behçet’s disease (BD), Systemic Lupus Erythematosus (SLE). In addition, it has been proposed that cases with very early onset in childhood can be underpinned by rare monogenic defects of immunity, which may require targeted treatments. Thus, subjects with early onset recurrent aphthous stomatitis receiving a clinical diagnosis of BD-like or SLE-like disease may deserve a further diagnostic workout, including immunologic and genetic investigations.

**Objective:**

To investigate how an immunologic, genetic and transcriptomics assessment of interferon inflammation may improve diagnosis and care in children with recurrent aphthous stomatitis with systemic inflammation.

**Methods:**

Subjects referred to the pediatric rheumatologist for recurrent aphthous stomatitis associated with signs of systemic inflammation from January 2015 to January 2020 were enrolled in the study and underwent analysis of peripheral lymphocyte subsets, sequencing of a 17-genes panel and measure of interferon score.

**Results:**

We enrolled 15 subjects (12 females, median age at disease onset 4 years). The clinical diagnosis was BD in 8, incomplete BD in 5, BD/SLE overlap in 1, SLE in 1. Pathogenic genetic variants were detected in 3 patients, respectively 2 *STAT1* gain of function variants in two patients classified as BD/SLE overlap and SLE, and 1 *TNFAIP3* mutation (A20 haploinsufficiency) in patients with BD. Moreover 2 likely pathogenic variants were identified in *DNASE1L3* and *PTPN22,* both in patients with incomplete BD. Interferon score was high in the two patients with *STAT1* GOF mutations, in the patient with *TNFAIP3* mutation, and in 3 genetic-negative subjects. In two patients, the treatment was modified based on genetic results.

**Conclusions:**

Although recurrent aphthous stomatitis associated with systemic inflammation may lead to a clinical diagnosis of BD or SLE, subjects with early disease onset in childhood deserve genetic investigation for rare monogenic disorders. A wider genetic panel may help disclosing the genetic background in the subset of children with increased interferon score, who tested negative in this study.

**Supplementary Information:**

The online version contains supplementary material available at 10.1186/s12969-021-00552-y.

## Background

Recurrent aphthous stomatitis (RAS) is a common and usually isolated and benign complaint in children. However, some cases of RAS present in association with other clinical symptoms or with signs of systemic inflammation, raising a diagnostic challenge to pediatricians. The most common form of RAS with systemic inflammation in pediatrics can be encountered in periodic fever, aphthous stomatitis, pharyngitis, and adenitis syndrome (PFAPA) [1]. The typical periodic recurrence of fever bouts with complete well-being between episodes, and with the absence of significant intestinal or musculoskeletal symptoms, makes PFAPA syndrome an easy diagnosis [2]. Although oral ulcers may present along with PFAPA crises, they are usually small-sized and rapidly self-recovering, never being the patient’s major complaint. Aphthous stomatitis accompanying recurrent fevers may also be part of rare syndromes like mevalonate kinase deficiency and Behçet’s disease (BD) [3, 4]. In these cases, aphthosis is often severe with multiple painful ulcerations and with slow recovery. RAS associated with raised acute phase reactants can also occur without a clinical story of recurrent fever and in these cases, it should raise the suspicion of inflammatory bowel disease (IBD) [5] or of a rheumatologic condition such as Systemic Lupus Erythematosus (SLE) [6] and BD [4]. However, RAS with systemic inflammation can also be a sign of an immunodeficiency, like HIV infection, neutropenia and monogenic mimics of BD [3, 7–14]. In a recent study, a significant proportion of children with atypical BD was found to have pathogenic mutations in genes associated with monogenic autoinflammatory disorders [15]. Similarly, monogenic disorders can be diagnosed in children who meet the classification criteria for SLE and present recurrent oral ulceration [16–18]. Some of these disorders are associated with dysregulated interferon (IFN)-driven inflammation, which can be revealed by the transcriptomic screening of the IFN signature [19–21]. Of note, an increased IFN signature may be found both in SLE and in BD [22], supporting that rare monogenic conditions with IFN-mediated inflammation may present in an area of clinical and pathological overlap between the two conditions.

The diagnostic challenge is even harder if we consider that some patients with SLE may also meet the classification criteria for pediatric BD [23].

Thus, we decided to investigate if a diagnostic protocol based on immunologic, transcriptomic and next generation sequencing analysis can contribute to improve the classification of subjects with recurrent oral ulcers and systemic inflammation, with possible benefits on the care of the patient in terms of meaningful therapeutic choices and complication surveillance.

## Patients and methods

### Patients

Subjects consecutively referred to the rheumatology unit of the IRCCS Burlo Garofolo with RAS from January 2015 to January 2020 were enrolled in the study.

As major inclusion criteria were considered the onset before 18 years of age and RAS. Moreover, patients were eligible to be enrolled in the study if they presented at least one of the following signs supportive of systemic inflammation: repeatedly elevated C reactive protein, raised erythrocyte sedimentation rate, genital ulcers, ocular inflammation, arthritis, hepatitis, vasculitis, skin rashes or abscesses. Exclusion criteria were considered: diagnosis of PFAPA, previous diagnosis of a familial periodic fever syndrome, and any refusal to participate in the study by the patient or parents.

For participating in the study, a signed informed consent was obtained from patients’ parents/guardians, according to an IRB approved protocol (RC 24/17). All subjects underwent peripheral blood sampling to analyze lymphocyte subsets and to obtain DNA and RNA for genomic and transcriptomic studies. Clinical and laboratory data were recorded in a structured anonymized database.

As regards the initial clinical diagnosis, the case was referred as to BD when at least three out of six criteria in the pediatric Behçet’s Disease (PEDBD) set were met, namely: at least 3 attacks/year of oral aphthosis; genital ulceration typically with scar; skin involvement with folliculitis/acne or erythema nodosum; ocular involvement with uveitis or retinal vasculitis; neurological signs with the exception of isolated headaches; vascular signs as thrombosis or aneurysms [23]. Subjects meeting only 2/6 criteria in PEDBD were defined, for the purposes of this study, as possible or incomplete BD (Additional file 2: Table S2).

Systemic Lupus Erythematosus was classified according to the 1997 revision of ACR criteria [24]. We did not refer to the new EULAR/ACR recommendations for SLE classification [25] since they have not yet been validated in children (Additional file 2: Table S3 and S4).

### Flow Cytometry

#### Immunophenotypic screening for major immune defects

Immunophenotype was performed on heparinized peripheral blood samples obtained from each patient by means of flow cytometry. 100 μL of blood were stained with two multicolour antibody panels in order to evaluate different lymphocyte subsets, including B cell subpopulations (naïve follicular B cells, marginal zone B cells, switched memory B cells and transitional B cells) and recent thymic emigrants (RTE).

Panel for B cells analysis contained the following antibodies: anti-CD45, anti-CD19, anti-CD38 (Becton Dickinson), anti-CD27, anti-IgM, anti-IgG, anti-CD21, anti-CD10 and anti-IgD (Miltenyi Biotec).

Panel for RTE analysis contained the following antibodies: anti-CD45, anti-CD19, anti-CD4, anti-CD8 (Becton Dickinson), anti-CD3, anti-CD45RA, anti-CD31, anti-CD16 and anti-CD56 (Miltenyi Biotec).

Samples were acquired with MACSQuant Analyzer 10 (Miltenyi Biotec) and analyzed with FlowLogic software (version 7.2.1, Inivai Technologies).

#### Expression of STAT1 in resting and stimulated peripheral blood cells

Peripheral blood mononuclear cells (PBMC), obtained from heparinized blood samples by means of density gradient centrifugation, were left unstimulated or were treated with 300 U/ml of IFN-alpha2a (Miltenyi Biotec). After 24 h of incubation in 5% CO_2_ at 37 °C cells were recovered, stained with antibodies to cell surface antigens (CD45 (Becton Dickinson), CD3 (Miltenyi Biotec), CD4 (Becton Dickinson)) fixed and permeabilized. PBMC were finally stained with anti-STAT1 antibody (Becton Dickinson) or with an isotype control antibody (Biolegend). Samples were acquired with MACSQuant Analyzer 10 (Miltenyi Biotec) and analyzed with FlowLogic software (version 7.2.1, Inivai Technologies).

### IFN signature and IFN score

Peripheral blood was collected in PAXgene Blood RNA Tubes (PreAnalytiX), extracted with PAXgene Blood RNA Kit (PreAnalytiX) following the manufacturer’s instructions, quantified with NanoDrop 2000 Spectrophotometer (Thermo Fisher Scientific) and retro-transcribed using SensiFAST cDNA Synthesis Kit (Bioline).

IFN signature was assessed by calculating the expression of a set of six IFN stimulated genes (*IFI27*, *IFI44L*, *IFIT1*, *ISG15*, *RSAD2* and *SIGLEC1*), as described elsewhere [26]. Briefly, Real-Time PCR was performed in the AB 7500 Real-Time PCR System (Applied Biosystems), using TaqMan Gene Expression Master Mix (Applied Biosystems) and UPL Probes (Roche). Gene expression was normalized to the amount of reference genes (*G6PD* and *HPRT1*) and quantified in respect to a calibrator sample (mix of ten healthy controls) using the 2^−ΔΔCt^ method. The IFN score (IS) provides the intensity of IFN signature, and it’s calculated as the median fold change of the six target genes. IS above 2.466 were considered positive, as previously calculated by Rice et al. to discriminate subjects with IFN-driven monogenic disorders [27].

### Genetic analysis

A custom-made panel for targeted gene panels sequencing (Thermo Fisher Scientific) was designed, including 17 genes listed below: *TNFAIP3* (NM_001270508 → NP_001257437), *STAT1* (NM_007315 → NP_009330), *DNASE2* (NM_001375 → NP_001366), *DNASE1L3* (NM_004944 → NP_004935), *TMEM173* (NM_198282 → NP_938023), *PRKCD* (NM_006254 → NP_006245), *TREX1* (NM_033629 → NP_338599), *SAMHD1* (NM_015474 → NP_056289), *IFIH1* (NM_022168 → NP_071451), *DNASE1* (NM_005223 → NP_005214), *ISG15* (NM_005101 → NP_005092), *PTPN22* (NM_015967 → NP_057051), *CTLA4* (NM_005214 → NP_005205), *STAT4* (NM_003151 → NP_003142), *TLR4* (NM_138554 → NP_612564), *RORC* (NM_005060 → NP_005051), *RC3H1* (NM_001300850 → NP_001287779).

Gene selection was based on lists of genes known to be associated with SLE and/or type I monogenic interferonopathies, BD or BD like disease (OMIM, Orphanet andPubMed databases).

All exons and flanking introns are covered 100%, the panel size is 62.58 kb and 2 pools of primers overlay the entire coding region referred to by the Human hg38 genome. DNA libraries were generated using Ion Ampliseq Library kit 2.0 (Thermo Fisher Scientific) according to manufacturer’s protocol, purified with magnetic bead technology Agencourt AMPure XP (Beckman Coulter) and quantified with KAPA Library Quantification Kits (Roche). The sequencing step was performed on the Ion TorrentTM PGM platform (Thermo Fisher Scientific). The signal processing was analyzed by the Torrent SuiteTM software v5.12 and the output file was further annotated using wANNOVAR free software (http://wannovar.wglab.org/).

All the obtained variants were filtered according the following criteria: minor allele frequency (MAF) (< 0.02 if recessive inheritance model or < 0.001 if dominant inheritance model), type of mutation (non-synonymous, nonsense, frameshift, splicing about 10 nucleotides from the splice site), damaging prediction and phenotype correlation.

Database used for MAF prediction was gnomAD browser (https://gnomad.broadinstitute.org/). In case of missense mutations, we considered those presenting a pathogenic prediction in at least two of the four in silico prediction tools Polyphen-2, SIFT, LRT and Mutation Taster, in addition to a high CADD score (> 15) and GERP score (Genomic Evolutionary Rate Profiling) as a measure of the conservation of the genomic position [28–33]. Human Gene Mutation Database professional (HGMD) was used to define association with mutation and specific phenotype.

Variants considered to be causative were validated by Sanger Sequencing both in proband and parents, when available.

## Results

### Patients’ characteristics

We enrolled 15 subjects (12 females, median age at disease onset 4 years, range 0.3–15 years). Recurrent aphthous stomatitis was a presenting symptom in all patients. The initial diagnosis was BD in 8, incomplete Behҫet Disease (BD-i) in 5, BD/SLE overlap in 1, SLE in 1. Clinical characteristics are shown in Table 1, together with the results of laboratory investigations.

**Table 1:** Clinical and laboratory characteristics of patients

Patient	Sex	Age at enrollment (y)	Onset (y)	RAS/year	Other clinical characteristics	Laboratory	HLA B51	Pathergy	Familial history	Diagnosis	PED BD *	ACR*	ACR EULAR^§^	Current therapy	Immunephenotype	IFN score	AA change Gene
#1	F	17	2	10–12	Oral candidiasis, autoimmune thyroiditis and gastritis, hemolytic anemia, polyarthralgia	ESR always ↑ ANA+, dsDNA+, Anti-Gastric Parietal Cell Antibody+	–	–	GPA (mother)	BD/SLE overlap	1/6	4	18	HCQ	N Stimulated STAT1 ↑	29.8	N574T *STAT1*
#2	F	17	1.5	20–30	Skin pustulosis	CRP mildly ↑	+	–	no	BD-i	2/6	no	no	Thalidomide	N	1.3	T109N *PTPN22*
#3	F	7	0.5	16	Genital ulcers, recurrent fever, skin pustulosis, arthralgias	ESR always ↑, CRP mildly ↑	+	–	RAS (aunt), chilblains (father)	BD	3/6	no	no	Colchicine, HCQ, topical clindamycin GC	N	2.4	no
#4	F	18	0.3	1	Genital ulcers, pustulosis, recurrent fever	ESR ↑, IgA ↑	+	–	no	BD	3/6	no	no	Colchicine, GC	N	0.4	no
#5	F	13	5	6	Necrotic folliculitis with skin ulcers, recurrent fever (no benefit from tonsillectomy)	CRP mildly ↑	+	n.d.	BD (mother)	BD-i	2/6	no	no	Colchicine, topical clindamycin	NK ↑B cells ↓	0.7	T971fs*2 *DNASE1L3*
#6	M	20	4	16	Abdominal pain, arthralgias, genital ulcers, pustulosis	ESR ↑, CRP ↑, IgA ↑	+	+	no	BD	3/6	no	no	Thalidomide	N	1.7	no
#7	F	24	12	14	Genital ulcers, folliculitis, arthralgias	ESR ↑	+	n.d.	no	BD	3/6	no	no	Colchicine	N	0.3	no
#8	F	15	11	12	Genital ulcers, abdominal pain, fatigue	ESR ↑, ANA-	n.d.	–	no	BD-i	2/6	no	no	Colchicine, GC	N	13.3	no
#9	F	13	3	4	Genital ulcers, arthralgias, aortic vasculitis	ESR ↑	+	+	RAS (mother)	BD	3/6	no	no	Colchicine	N	3.1	no
#10	F	9	6	9	Oral candidiasis, Hepatitis, recurrent paronychia	ESR ↑, ANA+, dsDNA+, Direct Coombs+, Normal complement	–	n.d.	no	SLE	no	4	15	MMF Colchicine	N, Basal and stimulated STAT1 ↑	13.5	T288A *STAT1*
#11	F	23	15	24	Erythema nodosum, uveitis, intestinal ulcerations	ESR ↑, IgA ↑	–	+	no	BD	4/6	no	no	ADA	N	1.5	no
#12	F	12	10	4	Recurrent HSP, lichen vulvar, IgA nephropathy	ESR ↑	+	–	RAS and IgA nephropathy (mother)	BD-i	2/6	no	no	Omega 3 fatty acids	n.d.	19.1	no
#13	M	17	11	4	Skin ulcers, vein thrombosis, fever (Hughes Stovin Syndrome)	CRP ↑	+	–	no	BD	3/6	no	no	ADA Apremilast	N	0.2	no
#14	F	25	0.5	6	Genital ulcerations	ESR ↑	–	–	no	BD-i	2/6	no	no	GC	N	1.2	no
#15	M	16	0.3	24	Fever, perianal ulcerations, skin abscesses, abdominal pain; no benefit from tonsillectomy	ESR ↑, usually normal CRP Fecal calprotectin ↑	–	–	BD (mother grandmother)	BD	3/6	no	no	GC, ADA started after genetic diagnosis	N	9.4	Q379X *TNFAIP3*

Legend: study results are reported in the last three columns. *RAS* recurrent aphthous stomatitis; *BD and BD-i* Behçet’s Disease and incomplete Behçet’s Disease; *GPA* Granulomatosis with polyangiitis; *CRP* C reactive protein; *ESR* erythrocyte sedimentation rate; *ANA* antinuclear antibodies; *dsDNA* antibodies to double stranded DNA; *HCQ* Hydroxychloroquine; *MMF* mycophenolate mofetil; *GC* glucocorticoids; *ADA* adalimumab; *HSP* Henoch-Schönlein purpura; *N* normal; “-“: negative result;” +”: positive result; *n.d.* not done; *: criteria items; §: score

*PEDBD* Consensus classification of Pediatric Behçet’s disease. Diagnosis of BD is performed with three of six items, whereas two of six items classify a patient as incomplete BD (BD-i). *ACR* American College of Rheumatology. To classify a patient with SLE, 4 criteria are required, either serially or simultaneously. *ACR-EULAR* American College of Rheumatology and the European League Against Rheumatism. ANA at a titer > = 1:80 on Hep-2 cells or an equivalent positive test (ever) is the entry condition. SLE classification requires at least one clinical criteria and ≧ 10 points are (Additional file 2: Table S2, S3 and S4)

### Immunophenotypic analysis

Immunophenotypic analysis showed only mild alterations of uncertain significance in subject #5, who showed an increased percentage of NK cells (23.3%) and low B cells (5.0%). No significant alteration was found in the other patients as concerns the main lymphocyte subset, including B cell subpopulations and recent thymic emigrants (RTE) (Additional file 1: Table S1).

### Genetic analysis

Pathogenic variants were detected in 3 patients, respectively 2 gain of function (GOF) mutation in *STAT1* (#1 with BD/SLE overlap, #10 with SLE) and 1 *TNFAIP3* mutation (A20 haploinsufficiency, #15 with BD). Additionally, 2 likely pathogenic variants were identified respectively in *DNASE1L3* (#5, BD-i) and *PTPN22* (#2 with BD-i) genes (Table 2).

**Table 2:** Genetic characteristics of identified mutations

Pt	Gene	Inheritance	Chr	Variant	dbSNP (Reference)	gnomAD	GERP	CADD	SIFT	PP2	LRT	MT	STATUS (carrier)
#1	*STAT1*	AD	2q32.2	c.1721A > C p.N574T	na	na	5.73	22.7	T	D	D	D	HZ (De novo)
#2	*PTPN22*	AD/mt	1p13.2	c.326C > A p.T109N	rs771337900	0.00001	5.41	27.2	D	D	D	D	HZ (mother)
#5	*DNASE1L3*	AR	3p14.3	c.288_289delCA p.T97Ifs*2	rs751206379 [34, 35]	0.00008	na	na	na	na	na	na	HZ (na)
#10	*STAT1*	AD	2q32.2	c.862A > G p.T288A	rs387906765 [36]	na	4.5	23.1	D	P	N	D	HZ (De novo)
#15	*TNFAIP3*	AD	6q23.3	c.C1135T p.Q379X	na	na	5.63	39	na	na	D	D	HZ (mother)

Legend. gnomAD refers to total allele frequency (exomes and genomes); *AD* autosomal dominant; *AR* autosomal recessive; *mt* multifactorial disease; *GERP* Genomic Evolutionary Rate Profiling; *CADD* Combined Annotation-Dependent Depletion; *SIFT* Sorting Intolerant From Tolerant; *PP2* Polyphen-2; *LRT* Likelihood Ratio Test; *MT* Mutation Taster; *T* tolerated; *D* damaging/deleterious; *P* possibly damaging; *N* neutral; *HZ* indicate heterozygous condition; *na* not available

The “variant” column shows cDNA sequence and Protein (amino acid) change referring to the coordinates of the gene transcript reported in the materials and methods

**Fig. 1:**
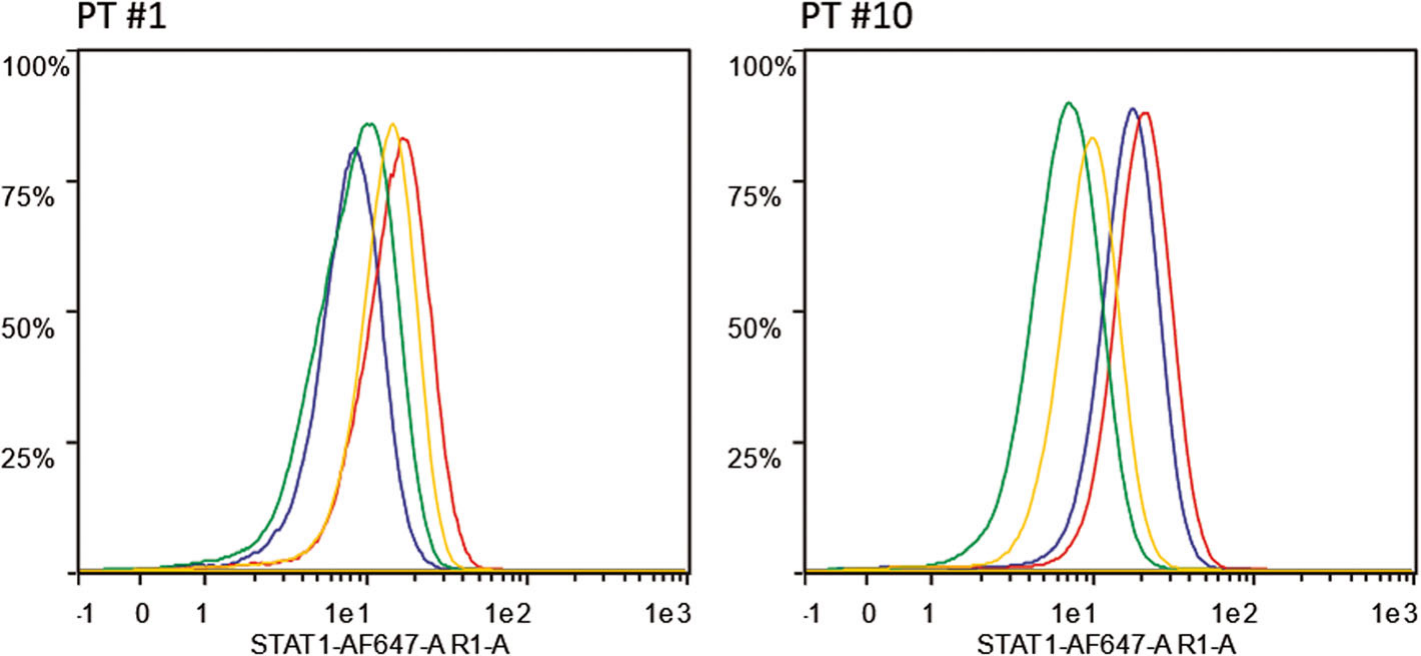
STAT1 expression. STAT1 expression in CD4 T cells from patient #1 and #10. Blue and Red: resting and IFN-alpha stimulated cells from patients; Green and Yellow: resting and IFN-alpha stimulated cells from healthy controls

The pathogenic role of variants was assessed by online bioinformatic tools and, in the case of *STAT1* GOF variants was also studied by the analysis of expression of the protein in peripheral blood cells. As shown in Fig. 1, patient #1 did not display a clear increase in STAT1 expression compared with the control. However, he carried the p.N574T variant, which lies in the same amino acid position previously described in association to a gain of function mutation of the protein and is similarly predicted to have a pathogenic effect [37]. Patient #10, who had the previously reported mutation p.T288A [36], showed a constitutive hyperexpression of STAT1 that was only slightly increased by IFN-alpha stimulation.

### IFN score

**Fig. 2:**
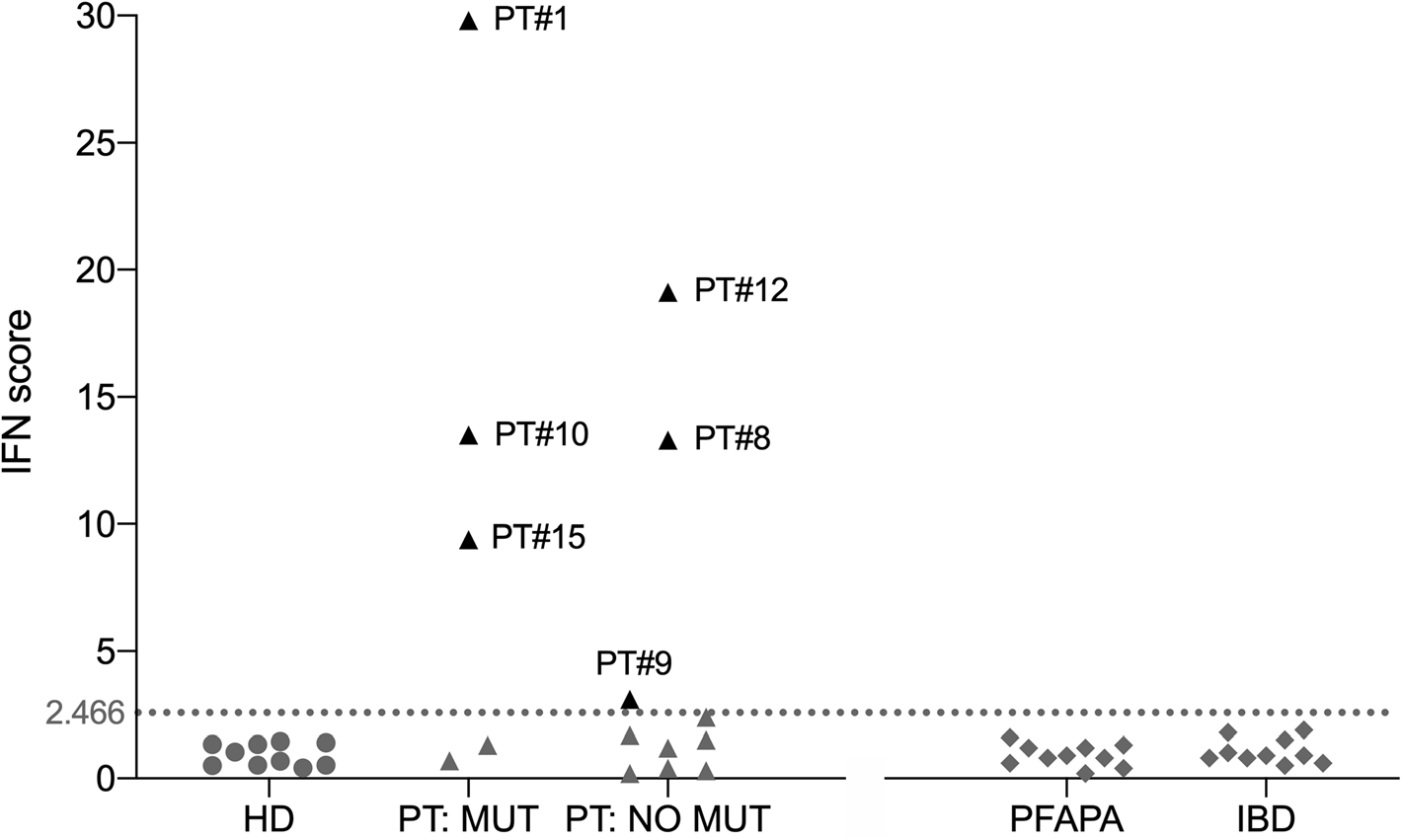
IFN score. IFN score (IS) in healthy controls (HC) and patients (PT), compared with subjects with PFAPA and IBD. The graphical display of IS is grouped in HC (n. 10, grey circle), patients (positive IS: black triangle; negative IS: grey triangle), with (“PT: MUT”, n. 5) and without genetic mutations (“PT: NO MUT”, n. 10), and subjects with PFAPA (n. 10, grey rhombus) and IBD (n. 10, grey rhombus). The dashed light-grey line represents the cut-off value (2.466) determined by Rice et al. [27]

IFN score was above the positivity cut-off in 6 subjects. Results are compared with measures in 20 subjects with stomatitis associated with non-monogenic conditions (10 patients with PFAPA and 10 with IBD), which tested negative. Two of them were classified as SLE (#10) or BD/SLE overlap (#1), 4 as BD (#9, #15) or incomplete BD (#8, #12) (Table 2). Three patients among those with high IS had positive genetic results (#1 and #10 with *STAT1* GOF mutations, #15 with *TNFAIP3* mutation, Fig. 2).

### Clinical follow-up in subjects diagnosed with a monogenic disorder

The results from this study affected the follow up of patients for various aspects. In two patients, previously classified as affected with SLE or BD/SLE overlap (#1 and #10), the diagnosis was changed in *STAT1* GOF disease and in one patient (#15), previously diagnosed with BD, the diagnosis was changed in A20 haploinsufficiency.

Based on the genetic results and on the presence of a high IS in these three patients, a treatment with JAK inhibitor could be reasonable, and indeed patient #10 started low dose baricitinib, in addition to mofetil mycophenolate and colchicine. After 1 month of treatment, mycophenolate was discontinued and in the following 2 months she didn’t experience recurrence of infections of aphthous stomatitis.

In the two patients with *STAT1* GOF, at follow-up more attention was put to the prevention and early treatment of infections, considering that they can present a severe course in this syndrome.

In patient #15, with A20 haploinsufficiency, a treatment with adalimumab was started, considering the high risks of disease progression without treatment and the colchicine intolerance. At the last follow-up, after 6 months of treatment, the patient didn’t refer further recurrence of aphthae, abscesses and fever crisis, even if laboratory investigations still revealed an increased IS. Of note, the patient #15 mother, also carrying the A20 mutation, was also affected with BD not requiring specific medications, whilst a treatment with adalimumab is being considered for the grandmother, who presents complaints similar to the nephew.

The follow-up care of patients carrying mutation in *DNASE1L3* and *PTPN22* so far was not affected by the genetic diagnosis.

Similarly, the finding of increased IS in other three patients without genetic mutations had so far, no obvious consequence on their clinical care.

## Discussion

Recurrent aphthous stomatitis (RAS) in children is usually a benign idiopathic disorder requiring only supportive care. However, in some cases RAS may be a presenting sign of a systemic disorder.

Patients may seek the attention of a rheumatologist because of early onset of complaints in infancy, high frequency of episodes, severity of oral ulcers, or due to the presence of a systemic inflammatory involvement, as revealed by laboratory examinations of by inflammatory symptoms involving other organs (such as genital ulcers, vasculitis, hepatitis, skin rashes and abscesses). As discussed further, these patients should undergo a clinical whole exome sequencing targeted to immune dysregulation genes.

The most common diagnosis in children with RAS and systemic inflammation is probably PFAPA syndrome [38]. However, RAS is rarely a serious issue in patients with PFAPA, where mouth ulcers are small, shallow, scattered or solitary, seemingly not painful; and frequently only are noted in episodes subsequent to the query; in contrast with other autoinflammatory syndrome where RAS is usually symptomatic and associated with systemic symptoms. In PFAPA genital ulcers are exceptionally reported, and the prognosis is usually good with prompt response to on demand glucocorticoids and complete healing with growth or after tonsillectomy [39]. Relapsing after tonsillectomy is a rare event, which may raise the suspicion of distinct disorders, such as Hereditary Periodic Fever syndromes [40, 41], or which may forecast a progression toward BD, as suggested by retrospective interviews performed in adulthood [42]. For this reason, we excluded subjects with PFAPA from our study, but included patients with a PFAPA-like onset whose disease progressed after tonsillectomy. Apart from PFAPA, the most common cause of RAS and inflammation is BD [43]. In these cases, oral and genital ulcerations are often a serious complaint. The positive pathergy test can help in the BD diagnosis, but it is not considered a criterion because in some populations, as in our series, can be infrequent. However, BD is quite rare in pediatrics and the onset of the disease in younger children should raise the suspicion of an underlying genetic disorder [4]. In agreement, a recent study in a pediatric series of BD showed that a high proportion of patients carried pathogenic mutations in genes associated with monogenic autoinflammatory disorders [15]. In our series, all cases carrying a pathogenic mutation in the analyzed genes had a disease onset before the age of 6 years, with an average age at onset of 3 years, which is very young and below the mean age of onset for BD (7 years) or SLE (11 years). Indeed, early onset may identify a subset of disease with severe phenotype which is likely to be enriched for genetic variants.

RAS with systemic inflammation may also be found in children with SLE. In these cases, too, a stronger genetic component may be found compared with adult-onset disease. For example, a recent Israeli study based on whole exome sequencing in childhood-onset SLE, showed that a high proportion of subjects carried pathogenic mutations associated with monogenic SLE [18]. Of note, there is a considerable overlap between some cases of pediatric SLE and BD and accordingly some of the causative genes may underlie both conditions, as in the case of *STAT1* gain of function mutations (GOF) and A20 haploinsufficiency [44].

Interestingly, oral ulceration associated with SLE is usually painless, multiple, nonspecific, and most common sites are tongue and labial mucosa.

In our series we detected two cases as well with *STAT1* GOF mutation. As expected, both patients had complained of mucosal candidiasis, which should have raised the suspicion of a primary immunodeficiency like *STAT1* GOF. However, in both cases, candidiasis was initially just considered as a complication of mucositis. Indeed, candida infections have been widely reported in association with SLE mucositis, even if we cannot know if any of the cases described in the literature were actually due to *STAT1* GOF [45–49, 50, 51]. Interestingly, one of our patients with *STAT1* GOF had received a diagnosis of SLE, meeting the ACR classification criteria, whilst the other one had a diagnosis of BD/SLE overlap disorder. The patient with A20 haploinsufficiency had a diagnosis of BD based on PEDDB criteria, but he also had some features more typical of SLE, such as increased IFN signature.

In typical cases, diagnosis of BD or SLE is a process driven by the clinical rheumatologist experience taking into account clinical and laboratory items. In atypical cases, classification criteria can be used to reinforce the diagnosis. However, there is general agreement that both diseases are heterogeneous entities encompassing distinct disorders characterized by systemic autoimmunity. It is still not clear whether the detection of monogenic forms accounting for particular phenotypes will reflect on better therapeutic choices and improved follow-up care in these cases.

In our series, the two patients with *STAT1* GOF had a partial control of the disease on treatment with mycophenolate mofetil and colchicine in the first case and with hydroxychloroquine in the second one. However, after the genetic diagnosis, low dose baricitinib was proposed to both patients to improve the control of symptoms. Moreover, given the high risk of severe infections in *STAT1* GOF disease, more attention was paid on the need for treating any bacterial or fungal infection with systemic antifungal or antibiotic therapy.

In the patient with A20 haploinsufficiency, who had a recurrent-relapsing disorder since his first years of life, a treatment with adalimumab was started with the aim of stopping inflammatory recurrence, as treatment with colchicine was refused because of intolerance. Of note, according to the literature, A20 haploinsufficiency may have distinctive clinical features compared with BD, such as early-onset, familial occurrence, recurrent fever attacks, gastrointestinal involvement, and infrequent ocular involvement, which can influence follow up and therapeutic choices [52].

It is noteworthy that the patient with A20 haploinsufficiency, similarly to other three subjects with BD or incomplete BD, had a high IS. Indeed, A20 haploinsufficiency may present some clinical overlap with SLE, which is more typically associated with increased IS [53]. For example, antinuclear and anti-DNA antibodies can be measured in almost half patients with A20 haploinsufficiency [54]. Moreover, a recent study showed that among subjects with A20 haploinsufficiency, those with a high IS may have a greater benefit from treatment with JAK inhibitors [20]. On the contrary, the role of IFNs in sporadic BD is less defined. Even if increased IFN production can be observed in a proportion of monocytes from patients with BD [55], IFN-mediated inflammation is not actually a common finding in BD. This concept is even reinforced by the notion that patients with BD uveitis may be treated with the administration of IFN-alpha. Thus, whether a high IS may identify a particular disease subset in BD, is an interesting issue to be addressed in future studies, with a wider set of genes to be investigated.

Clinical pictures overlapping distinct rheumatologic conditions are common in adults but are much rarer in pediatrics. Our experience supports the new paradigm that every child with an overlapping rheumatological phenotype should be studied for a rare monogenic condition.

In addition to *STAT1* and *TNFAIP3*, we found also likely pathogenic variants in *PTPN22* and *DNASE1L3* in two patients with incomplete BD. Although *PTPN22* polymorphisms have been considered as susceptibility factors for BD or SLE, data are controversial and may be affected by distinct population settings [56, 57]. Biallelic DNASE1L3 deficiency is associated with early-onset familial SLE [58, 59] and hypocomplementemic urticarial vasculitis syndrome [34, 60]. Heterozygous hypomorphic mutations in *DNASE1L3* have been described in subjects with SLE anti-DNA antibodies sensitive to DNASE1L3 digestion [35]. However, no sign of SLE was present in our patient and the association with a BD-like phenotype in our case is of unclear significance.

The high percentage of monogenic cases in our cohort, is probably due to a bias in patient selection. Our Institute is considered one of the national research centers of rare diseases that attracts complex cases not solved in other institutes.

Is also important underline that we did not identify any patient with RAS and inflammatory bowel disease; this is probably a referral bias, notably three of patients reported were previously referred to our Gastroenterology unit.

## Conclusions

RAS with systemic inflammation may lead to a clinical diagnosis of BD or SLE, moreover patients with early disease onset deserve genetic investigation for rare monogenic disorders. Pediatricians should be aware that RAS can drive the suspicion of a monogenic disorder diagnosis if onset is very early and there are signs of organ involvement that cannot be attributed certainty to a specific polygenic disease.

The IS can be elevated in a subset of patients with BD who sometimes also present clinical elements of overlap with SLE. However, a wider genes panel should be investigated in the subset of children with increased IS but negative for genetic analyses. Integration of laboratory data can lead to a correct disease classification and significant improvements in therapy and follow-up.

## Supplementary Information

**Additional file 1.**

**Additional file 2.**

## Data Availability

The datasets used and/or analysed during the current study are available from the corresponding author on reasonable request.

## Abbreviations

BD: Behçet’s disease
BD-i: Incomplete Behçet’s disease
SLE: Systemic Lupus Erythematosus
RAS: Recurrent aphthous stomatitis
PFAPA: Periodic fever, aphthous stomatitis, pharyngitis and adenitis syndrome
IBD: Inflammatory bowel disease
GOF: Gain of function
