# Supplementary material for: Genetic and immunologic findings in children with recurrent aphthous stomatitis with systemic inflammation
Source: Pediatr Rheumatol Online J. 2021 May 10;19:70. doi: 10.1186/s12969-021-00552-y (PMC8111718; doi:10.1186/s12969-021-00552-y)
Supplement: Supplementary file 1 — Additional file 1. [file 12969_2021_552_MOESM1_ESM.docx]

**Additional file 1**

**Table S1.** Immunophenotyping data from 15 patients.

| Pt | CD3 (% CD45++ lymphocytes) [normal values %] | CD4/CD8 [normal values] | RTE (% CD4+ lymphocytes) [normal values %] | NK (% CD45++ lymphocytes) [normal values %] | CD19 (% CD45++ lymphocytes) [normal values %] | Transitional B cells (% B lymphocytes) [normal values %] | Naïve B cells (% B lymphocytes) [normal values %] | IgM Memory B cells (% B lymphocytes) [normal values %] | Switched memory B cells (% B lymphocytes) [normal values %] |
| --- | --- | --- | --- | --- | --- | --- | --- | --- | --- |
| # 1 | 70.1  [59.0-83.0] | 1.1  [1.4-4.0] | 29.1  [6.4-51.0] | 7.1  [6.0-27.0] | 18.8  [2.8-17.4] | 7.3  [0.6-3.0] | 69.3  [42.0-82.0] | 4.6  [1.7-29.3] | 9.7  [2.3-26.5] |
| # 2 | 74.4  [59.0-83.0] | 1.2  [1.4-4.0] | 17.4  [6.4-51.0] | 14.7  [6.0-27.0] | 8.7  [2.8-17.4] | 0.9  [0.6-3.0] | 67.9  [42.0-82.0] | 8.7  [1.7-29.3] | 11.4  [2.3-26.5] |
| # 3 | 66.7  [43.0-63.0] | 1.5  [1.0-3.0] | 57.6  [43.9-66.4] | 9.1  [4.0-17.0] | 23.1  [8.5-20.2] | 2.0  [3.4-9.0] | 69.7  [47.8-69.8] | 10.3  [6.3-22.0] | 12.5  [1,8-14,2] |
| # 4 | 72.6  [59.0-83.0] | 1.3  [1.4-4.0] | 34.3  [6.4-51.0] | 11.2  [6.0-27.0] | 14.6  [2.8-17.4] | 0.2  [0.6-3.0] | 53.5  [42.0-82.0] | 17.0  [1.7-29.3] | 26.1  [2.3-26.5] |
| # 5 | 67.8  [40.1-68.0] | 1.6  [1.5-3.7] | 45.7  [33.5-58.2] | 23.3  [3.0-22.0] | 5.0  [7.8-23.7] | 7.0  [1.5-7.3] | 65.3  [64.6-80.1] | 10.3  [4.7-22.2] | 11.5  [3.3-14.2] |
| # 6 | 60.5  [59.0-83.0] | 2.6  [1.4-4.0] | 25.9  [6.4-51.0] | 23.9  [6.0-27.0] | 15.4  [2.8-17.4] | 0.5  [0.6-3.0] | 85.8  [42.0-82.0] | 6.4  [1.7-29.3] | 2.8  [2.3-26.5] |
| # 7 | 76.0  [59.0-83.0] | 1.3  [1.4-4.0] | 48.9  [6.4-51.0] | 16.2  [6.0-27.0] | 6.8  [2.8-17.4] | 0.3  [0.6-3.0] | 53.3  [42.0-82.0] | 23.7  [1.7-29.3] | 20.0  [2.3-26.5] |
| # 8 | 74.9  [41.5-68.0] | 1.3  [1.4-4.0] | 46.2  [32.7-60.9] | 7.2  [3.0-22.0] | 13.8  [7.8-15.1] | 1.2  [1.5-7.3] | 82.7  [59.0-81.1] | 5.6  [4,6-23,8] | 7.8  [3,0-16,3] |
| # 9 | 71.6  [40.1-68.0] | 1.6  [1.5-3.7] | 65.8  [33.5-58.2] | 5.6  [3.0-22.0] | 20.9  [7.8-23.7] | 2.0  [1.5-7.3] | 78.8  [64.6-80.1] | 8.0  [4.7-22.2] | 6.8  [3.3-14.2] |
| # 10 | 84.3  [43.0-63.0] | 1.4  [0.9-2.5] | 49.2  [40.1-54.8] | 3.8  [4.0-17.0] | 8.0  [4.3-18.2] | 0.7  [2,9-9,0] | 85.8  [51,5-73,3] | 8.6  [6,5-22,2] | 2.5  [2,8-14,9] |
| # 11 | 67.1  [59.0-83.0] | 2.9  [1.4-4.0] | 45.5  [6.4-51.0] | 8.9  [6.0-27.0] | 20.9  [2.8-17.4] | 1.8  [0.6-3.0] | 76.8  [42.0-82.0] | 6.0  [1.7-29.3] | 8.9  [2.3-26.5] |
| # 12 | n.d. | n.d. | n.d. | n.d. | n.d. | n.d. | n.d. | n.d. | n.d. |
| # 13 | 67.0  [41.5-68.0] | 1.2  [1.4-4.0] | 30.0  [32.7-60.9] | 7.1  [3.0-22.0] | 22.4  [7.8-15.1] | 0.2  [1.5-7.3] | 63.8  [59.0-81.1] | 17.0  [4,6-23,8] | 13.9  [3,0-16,3] |
| # 14 | 68.8  [59.0-83.0] | 3.6  [1.4-4.0] | 31.2  [6.4-51.0] | 16.4  [6.0-27.0] | 12.5  [2.8-17.4] | 2.6  [0.6-3.0] | 72.2  [42.0-82.0] | 10.6  [1.7-29.3] | 10.0  [2.3-26.5] |
| # 15 | 66.7  [41.5-68.0] | 1.4  [1.4-4.0] | 24.5  [32.7-60.9] | 12.9  [3.0-22.0] | 17.0  [7.8-15.1] | 3.9  [1.5-7.3] | 81.3  [59.0-81.1] | 4.2  [4,6-23,8] | 8.2  [3,0-16,3] |
