# Supplementary material for: Genetic and immunologic findings in children with recurrent aphthous stomatitis with systemic inflammation
Source: Pediatr Rheumatol Online J. 2021 May 10;19:70. doi: 10.1186/s12969-021-00552-y (PMC8111718; doi:10.1186/s12969-021-00552-y)
Supplement: Supplementary file 2 — Additional file 2. [file 12969_2021_552_MOESM2_ESM.docx]

**Additional file 2**

**Table S2.** Consensus classification of pediatric Behçet’s disease (BD).

| **Item** | **Description** | **Value/item** |
| --- | --- | --- |
| Recurrent oral aphthosis | At least three attacks/year | 1 |
| Genital ulceration or aphthosis | Typically with scar | 1 |
| Skin involvement | Necrotic folliculitis, acneiform lesions, erythema nodosum | 1 |
| Ocular involvement | Anterior uveitis, posterior uveitis, retinal vasculitis | 1 |
| Neurological signs | With the exception of isolated headaches | 1 |
| Vascular signs | Venous thrombosis, arterial thrombosis, arterial aneurysm | 1 |

Three of six items are required to classify a patient as having pediatric BD. Two of six items classify a patient as incomplete BD (BD-i) [23].

**Table S3.** American College of Rheumatology revised criteria for the classification of systemic lupus erythematosus (SLE).

| **Criteria** | **Definition** |
| --- | --- |
| 1. Malar rash | Fixed erythema, flat or raised, over the malar eminences, tending to spare the nasolabial folds |
| 1. Discoid rash | Erythematous raised patches with adherent keratotic scaling and follicular plugging; atrophic scarring may occur in older lesions |
| 1. Photosensitivity | Skin rash as a result of unusual reaction to sunlight, by patient history or physician observation |
| 1. Oral ulcers | Oral or nasopharyngeal ulceration, usually painless, observed by physician |
| 1. Non-erosive arthritis | Involving 2 or more peripheral joints, characterised by tenderness, swelling or effusion |
| 1. Pleuritis or Pericarditis | - Pleuritis: convincing history of pleuritic pain or rubbing heard by physician or evidence of pleural effusion   OR   - Pericarditis: documented by electrocardiogram or rub or evidence of pericardial effusion |
| 1. Renal disorder | - Persistent proteinuria >0,5 grams per day or > than 3+ if quantization not performed   OR   - Cellular casts: may be red cells, hemoglobin, granular, tubular or mixed |
| 1. Neurologic disorder | - Seizures: in the absence of offending drugs or known metabolic derangements, e.g uremia, ketoacidosis or electrolyte imbalance   OR   - Psychosis: in the absence of offending drugs or known metabolic derangements, e.g uremia, ketoacidosis or electrolyte imbalance |
| 1. Hematologic disorder | - Hemolytic anemia: with reticulocytosis OR - Leukopenia <4000/mm^3^ on > = 2 occasions OR - Lymphopenia <1.500/mm^3^ on > = 2 occasions. OR - Thrombocytopenia <100.000/mm^3^ in absence of offending drugs |
| 1. Immunologic disorder | - Anti-DNA: antibody to native DNA in abnormal titer OR - Anti SM: presence of antibody to Sm nuclear antigen OR - Positive finding of antiphospholipid antibodies on:  1. An abnormal serum level of IgG or IgM anticardiolipin antibodies 2. A positive test result for Lupus anticoagulant using a standard method 3. A false positive test result for at least 6 months confirmed by Treponema Pallidum immobilization or fluorescent Treponema antibody absorption test. |
| 1. Positive anti-nuclear antibody | An abnormal titer of antinuclear antibody by immunofluorescence of an equivalent assay at any point in time and in the absence of drugs |

At least 4 criteria need to be met, either serially or simultaneously, for a patient to be classified as having SLE [24].

**Table S4.** American College of Rheumatology (ACR) and the European League Against Rheumatism (EULAR) criteria

Entry criterion: antinuclear antibodies (ANA) at a titer >= 1:80 on Hep-2 cells or an equivalent positive test (ever)

If absent, do not classify as SLE

If present, apply additive criteria. SLE classification requires at least one clinical criterion and ≧ 10 points (if more criteria are present in one domain, only the highest is counted) [25].

| **Clinical domains and criteria** | **Weight** | **Immunology domains and criteria** | **Weight** |
| --- | --- | --- | --- |
| ***Constitutional***  Fever | 2 | ***Antiphospholipid antibodies***  Anti-cardiolipin antibodies OR Anti-beta2GP1 antibodies OR Lupus anticoagulant | 2 |
| ***Hematologic***  Leukopenia  Thrombocytopenia  Autoimmune hemolysis | 3  4  4 | ***Complement proteins***  Low C3 or low C4  Low C3 and low C4 | 3  4 |
| ***Neuropsychiatric***  Delirium  Psychosis  Seizure | 2  3  5 | ***SLE-specific antibodies***  Anti-dsDNA antibody OR  Anti-Smith antibody | 6 |
| ***Mucocutaneous***  Non-scarring alopecia  Oral ulcers  Subacute cutaneous OR discoid lupus  Acute cutaneous lupus | 2  2  4  6 |  |  |
| ***Serosal***  Pleural or pericardial effusion  Acute pericarditis | 5  6 |  |  |
| ***Musculoskeletal***  Joint involvement | 6 |  |  |
| ***Renal***  Proteinuria >0.5g/24h  Renal biopsy Class II or V lupus nephritis  Renal biopsy Class III or IV lupus nephritis | 4  8  10 |  |  |
